# Factor Structure, Reliability, and Validity of the Japanese Version of the Disgust Propensity and Sensitivity Scale-Revised

**DOI:** 10.1371/journal.pone.0164630

**Published:** 2016-10-12

**Authors:** Kazunori Iwasa, Tsunehiko Tanaka, Yuki Yamada

**Affiliations:** 1 Department of Educational Psychology, Shujitsu University, Okayama, Japan; 2 Department of Community Psychiatric Medicine, Shiga University of Medical Science, Otsu, Shiga, Japan; 3 Faculty of Education, Niigata University, Niigata, Japan; 4 Faculty of Arts and Science, Kyushu University, Fukuoka, Japan; Kyoto University, JAPAN

## Abstract

The Disgust Propensity and Sensitivity Scale-Revised is one of the most widely used measures of individual differences for the emotion of disgust. It consists of 2 subscales: disgust propensity and disgust sensitivity. This study examined the factor structure, reliability, and validity of the Japanese version of the revised Disgust Propensity and Sensitivity Scale. Japanese participants (N = 1067) completed the scale as well as the Padua Inventory, Anxiety Sensitivity Index, State-Trait Anxiety Inventory, and Positive and Negative Affective Schedule. The participants were divided into 3 samples: Sample 1 (n = 481, mean age = 23.05, 186 males and 295 females); Sample 2 (n = 492, mean age = 20.27, 243 males and 249 females); and Sample 3 (n = 94, mean age = 22.68, 35 males and 58 females). We combined Samples 1 and 2 (n = 973, mean age = 21.66, 429 males and 544 females), and then created 2 subsamples to ensure the mutual independence of the samples used for two different factor analyses: subsample 1 (n = 486, mean age = 21.86, 199 male and 287 female) for exploratory factor analysis and subsample 2 (n = 487, mean age = 21.40, 230 male and 257 female) for confirmatory factor analysis. We examined test-retest reliability using Sample 3, and construct validity using Samples 1, 2, and the combined sample. Exploratory and confirmatory factor analyses revealed that the item-factor structure of the Japanese Disgust Propensity and Sensitivity Scale-Revised was identical to the English version. Moreover, the scale showed good internal consistency, test-retest reliability, and construct validity for empirical support as provided by correlational analyses. Results revealed adequate psychometric properties of the scale. This study provided the first examples of empirical support for the DPSS-R-J.

## Introduction

Disgust is one of the basic emotions and is characterized by a revulsion response towards potential contamination [1]. It induces a strong tendency to avoid stimuli that can elicit the disgust emotion. Disgust characteristics serve an adaptive function that works to avoid disease, and protect us from infection, contamination, or sickness caused by physical contact with noxious stimuli in spite of its aversive nature [2].

In the last three decades, disgust has attracted much interest from researchers in a diverse range of fields, especially in the fields of psychology and psychiatry [3]. A number of studies have examined the nature of disgust such as its developmental features [4], behavioral consequences [5, 6], and cognitive biases [7, 8]. They have shed light on the psychological nature of disgust. They also revealed that disgust affects emotional and behavioral problems, as well as psychopathologies like obsessive-compulsive disorders [9], post-traumatic stress disorder [10], animal phobia [11], blood-injection-injury phobia [12], eating disorders [13], borderline personality disorder [14], and so forth. Disgust seems to engage the development and maintenance of various psychopathologies. Furthermore, treatment implications that involve disgust have been proposed [9, 15, 16]. It is expected that these researches will uniquely contribute to the treatments of psychological problems and psychopathologies treatments.

In contrast to this progress, few studies have examined disgust in Japan. One of the reasons Japanese disgust research has not progressed sufficiently is the lack of a formal measurement tool for assessing individual characteristics; however, this situation can be resolved by developing a Japanese formal assessment tool that does exactly that. Such an assessment tool would enable the examination of how disgust plays a role in psychopathology like obsessive-compulsive disorders or phobia among the Japanese population. This kind of examination leads to a verification of the applicability of scientific knowledge concerning the role of disgust in psychopathology, as was found to be the case in other cultural spheres. Moreover, some previous studies have found cultural differences in experiences of disgust [17], although it is thought to be one of the basic emotions. The construction of a formal assessment tool for use with the Japanese population is necessary because it will enable investigations into the culturally specific characteristics of disgust in Japan, as well as the cultural universality of the emotion.

Disgust has been measured several different ways in the literature. These scales typically assess individual differences in disgust called “disgust sensitivity” and “disgust propensity.” Disgust sensitivity refers to how unpleasant the experience of disgust is to the individual. Disgust propensity refers to how easily the individual is disgusted in any given situation. Such definitions are similar to conceptualizations of individual differences on anxiety: disgust sensitivity corresponds to anxiety sensitivity (i.e., the fear of anxiety-related sensations), and disgust propensity corresponds to trait anxiety (i.e., the general tendency to experience anxiety symptoms in any given situation). In the field of anxiety research, trait anxiety and anxiety sensitivity are conceptually distinguished; however, they partially overlap [18, 19]. Furthermore, numerous studies have found unique effects of both anxiety differences on the development and stability of psychopathologies [20–23]. Based on these findings and conceptualizations concerning anxiety, distinguishing between disgust sensitivity and disgust propensity (i.e., trait disgust) is theoretically reasonable. According to previous studies, disgust propensity and sensitivity are either moderately or strongly correlated [24, 25], suggesting that these two concepts overlap. Given this assumption, the conceptual distinction between disgust propensity and sensitivity should be tested empirically in the same way it was in previous studies [24, 25]. In this study, we adopted the distinction between trait anxiety and anxiety sensitivity as the framework under which to examine the conceptual independence of disgust propensity and disgust sensitivity. Both disgust propensity and trait anxiety refer to the frequency of occurrence of negative affect, while both disgust sensitivity and anxiety sensitivity refer to fear or unpleasantness toward the symptoms and bodily sensations accompanying such negative affect. These conceptual similarities led us to assume that disgust propensity will correlate more closely with trait anxiety than anxiety sensitivity; however, disgust sensitivity will correlate more closely with anxiety sensitivity than trait anxiety. Testing these hypotheses would reveal whether disgust propensity and sensitivity are distinguishable from each other.

The Disgust Propensity and Sensitivity Scale (DPSS) is the measurement to assess both disgust sensitivity and disgust propensity simultaneously. This scale was originally developed by Cavanagh and Davey [26]. Initially, it had 32 items and covered 2 factors (i.e., disgust sensitivity and disgust propensity); however, a subsequent study found that the revised version of the scale (DPSS-R) that comprised 16 items for the 2 factors was statistically better [24]. The validity and reliability of the DPSS-R have been confirmed in several studies [24, 25, 27]. There are several versions of the DPSS-R containing different item-factor structures. Olatunji and colleagues specified an alternative factor structure that had the same number of items and factors as the DPSS-R; however, it differed in the item-factor structure [25]. Furthermore, Fergus and Valentiner developed a short version of the DPSS-R consisting of only 12 items and the 2 factors [27]. It is not clear which factor structure is more appropriate for the Japanese version. Therefore, to develop a Japanese version of the DPSS-R (DPSS-R-J), there is a need to investigate the model fitness of the factor structure based on the results of an exploratory factor analysis.

To investigate the construct validity of the DPSS-R-J, we created the following six hypotheses: (1) For convergent validity, the DPSS-R-J will show moderate to strong positive correlations with anxiety sensitivity and trait anxiety; (2) the DPSS-R-J will show moderate correlations with state anxiety and negative affect; (3) disgust sensitivity will correlate more with anxiety sensitivity than with trait anxiety; (4) disgust propensity will correlate more with trait anxiety than with anxiety sensitivity; and (5) for discriminant validity, the DPSS-R-J will have no association with positive affect. Furthermore, we investigated the relationship between obsessive-compulsive symptoms. In the literature, contamination-related washing obsessions and compulsions are linked more strongly to disgust than are other obsessive-compulsive symptoms [28]. Therefore, we predicted that (6) the DPSS-R-J will be moderately positively correlated with obsessive-compulsive symptomatology; specifically, it will more strongly correlate to the contamination symptom assessed by the dirt subscale of the Japanese version of the Padua Inventory than to the other symptoms. Above all, the primary purpose of this study is to specify a factor structure of the DPSS-R-J by both exploratory and confirmatory factor analyses. The secondary purpose is to test its construct validity and reliability.

## Methods

### Ethics Statement

This study was conducted after receiving approval from the ethical review board of Shiga Medical University, Japan (approval number: 2012-24-40), and the ethical committees of Kyushu University approved the protocol (approval number: 2013–008). Written informed consent was obtained from all participants. Participation was voluntary, and participants had the right to withdraw from the research at any time without providing a reason.

### Participants and Procedures

Participants were recruited between 2012 and 2013 from university classes and a community lecture meeting about general psychology. Participants consisted of Japanese undergraduate students, graduate students, and community members who lived in Hokkaido, Ibaraki, Tokyo, Aichi, Shiga, Osaka, Okayama, Tokushima, or Kumamoto. Participants did not receive a monetary reward.

In this study, we conducted three surveys. In the first survey (Sample 1), participants completed the DPSS-R-J, the Padua Inventory (PI), the State-Trait Anxiety Inventory-Trait (STAI-T), and the Anxiety Sensitivity Scale (ASI). In the second survey (Sample 2), participants completed the DPSS-R-J, PI, State-Trait Anxiety Scale-State (STAI-S), and the Positive and Negative Affect Schedule (PANAS). Data from the first and second survey were combined and used to test the factor structure and validity of the DPSS-R-J. In a third survey (Sample 3), participants completed the DPSS-R-J twice. The time interval between Time 1 and Time 2 was four weeks. Data from the third survey was used to estimate the test-retest reliability of the DPSS-R-J. In Sample 3, participants’ Time 1 and Time 2 data were matched using a written identification. Before administering each survey, one of the authors or the instructor explained the purpose of the study and addressed any ethical issues. We also distributed documents describing the purpose of the study and related ethical issues before distributing the questionnaires. A completed and submitted questionnaire was collected after obtaining written informed consent. We asked the participants whether they had any history of psychiatric disorders within the past year. Those who answered “yes” to this question were excluded them from the statistical analyses to the control potential influence of psychiatric disorders.

**Fig 1:**
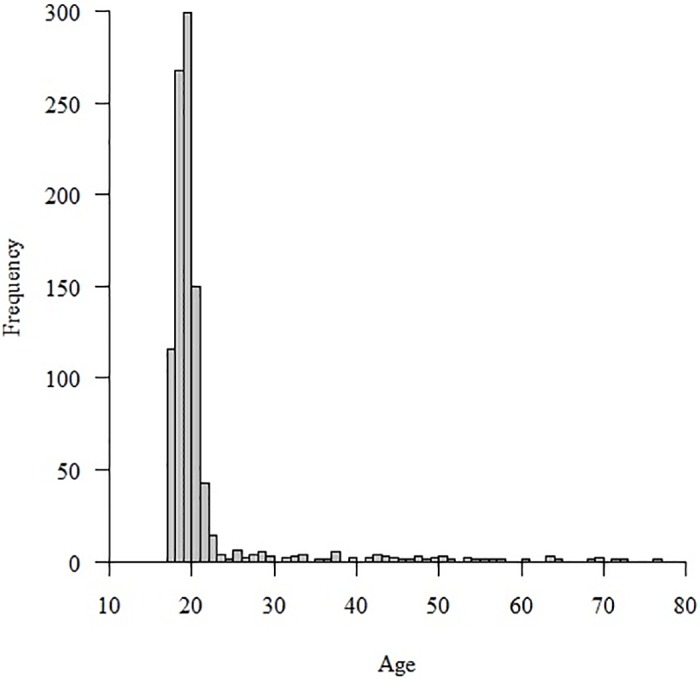
Age distribution of the combined sample (n = 973) in this study.

Sample 1 originally comprised 501 participants; however, 20 participants who self-reported a history of psychiatric disorders within the year were excluded from analyses. Therefore, Sample 1 comprised 481 participants (mean age = 23.05, range = 18–77, SD = 9.80) including 186 males and 295 females. Sample 2 originally comprised 511 participants; however, 19 participants who self-reported a history of psychiatric disorders within the year were excluded from analyses. Therefore, Sample 2 comprised 492 participants (mean age = 20.27, range = 18–64, SD = 3.63) including 243 males and 249 females. The combined sample of Sample 1 and 2 comprised 973 participants (mean age = 21.66, range = 18–77, SD = 7.48) including 429 male and 544 female. Fig 1 shows the age distribution of the combined sample. Sample 3 originally comprised 97 participants; however, 4 participants who self-reported a history of psychiatric disorders within the year were excluded from analyses. Therefore, Sample 3 comprised 93 participants (mean age = 22.68, range = 18–56, SD = 6.75) including 35 males and 58 females.

### Translation of the DPSS-R-J

Development of the DPSS-R-J was permitted by the first author of the original DPSS [26]. A forward/backward translation procedure was used to ensure equivalence between the original English version and the Japanese translated version. First, two native Japanese clinical psychologists who were fluent in both Japanese and English translated the original DPSS-R into Japanese independently. After comparing those two forward translations, we developed the first version of the DPSS-R-J. Second, this forward translation was then back translated into English by other translators who had no knowledge of the original DPSS-R. These translators were native English speakers who were proficient in Japanese. Third, along with a native English speaker (an English teacher who works at a Japanese university), we compared the English backward translation and original English version to assure that there were no differences in the meaning of the questions. After making minor amendments, we developed a provisional version of the DPSS-R-J. Fourth, we conducted a pilot test of the DPSS-R-J with 20 Japanese undergraduate students to ensure that the translation was fully understood by non-expert Japanese individuals. Consequently, we concluded that the translation accuracy of the DPSS-R-J was acceptable, and decided to use this version of the DPSS-R-J in this study.

### Measurements

All participants were asked to provide their age, sex, and presence or absence of psychiatric disorders history within a year before completing the following inventories.

#### Disgust Propensity and Sensitivity Scale (DPSS-R-J)

The DPSS-R-J consisted of 16 items designed to measure the frequency of disgust experience (disgust propensity) and the emotional impact of disgust experiences (disgust sensitivity), as does the DPSS-R. Both the disgust propensity and disgust sensitivity subscales included 8 items. Participants rated their agreement with each item on a 5-point Likert scale (from 1 “*never*” to 5 “*always*”). The fitness of the above-mentioned two-factor structure of the DPSS-R was acceptable [24]. Moreover, both the disgust propensity subscale (α = .78; test-retest reliability = .69) and disgust sensitivity subscale (α = .77; test-retest reliability = .77) were adequately reliable [24]. Previous studies reported women scored significantly higher than men on the DPSS-R, which is consistent with the findings of other measures of disgust [25, 29]. Such gender differences imply there are DPSS-R factor structure differences between men and women. In this study, we examine gender differences on the subscale scores of the DPSS-R-J. However, we will adopt the same factor structures for men and women even if gender differences are found, because the invariance of the factor structure between men and women damages the generalizability of findings using this scale.

#### Padua Inventory (PI)

The PI, which was originally composed by Sanavio, is a self-report measure designed to assess obsession-compulsive symptoms [30]. PI items are rated on 5-point Likert scale (from 0 “*not at all*” to 4 “*very much*”). In this study, we used the Japanese version of the PI (PI-J) that was developed by Sugiura [31]. The PI-J consisted of 60 items with the following 5 factors: doubt, impulse, check, dirt, and precision. This factor structure is somewhat different from the original version. The original version of PI also consisted of 60 items; however, it measures the following 4 factors: impaired control of mental activity, becoming contaminated, checking behavior, and urges/worries of losing control over motor behaviors. According to Sugiura [31], 4 of the 5 factors of the Japanese version are almost identical to the original 4 factors: doubt corresponds to impaired control of mental activity, impulse corresponds to urges/worries of losing control over motor behaviors, check corresponds to checking behavior, and dirt corresponds to becoming contaminated. The remaining factor (precision) was found by an exploratory factor analysis and was unique to the Japanese version. However, this added factor is similar to a factor involved in an alternative factor structure of the PI [32]. The reliability and validity of PI-J has been confirmed by Sugiura [31]. In this study, the internal consistencies of its 5 subscales were high (Cronbach’s α ranged from 0.84 to 0.93).

#### State-Trait Anxiety Inventory (STAI)

The STAI is a widely used inventory to assess individual differences in anxiety [33]. It contains two subscales assessing state anxiety and trait anxiety. Both subscales consisted of 20 items. STAI items are rated on 4-point Likert scale (from 1 “*not at all*” to 4 “*very much so*”). In this study, we used the Japanese version of STAI (STAI-J). The validity and reliability of this version have been demonstrated [34, 35]. In this study, the internal consistencies of both the state version (Cronbach’s α = 0.88) and the trait version (Cronbach’s α = 0.89) of STAI-J were adequate.

#### Anxiety Sensitivity Index (ASI)

The ASI is a 16-item measure designed to assess individual difference in anxiety sensitivity [36], and it has a single-factor structure. Anxiety sensitivity refers to a belief that the experience of anxiety/fear causes illness, embarrassment, or additional anxiety [36]; in other words, it causes a fear of anxiety-related symptoms. ASI items are rated on 5-point Likert scale (from 0 “*very little*” to 4 “*very much*”). In this study, we used the Japanese version of ASI (ASI-J). The ASI-J consisted of 16-items and a single-factor structure identical to the original version [37]. Its reliability and validity were confirmed in previous studies [37, 38]. In this study, the internal consistency of the ASI-J was adequate (Cronbach’s α = 0.90).

#### Positive and Negative Affect Schedule (PANAS)

The PANAS is a 20-item self-report measure of affective states composed by Watson, Clark, and Tellegen [39]. It has two subscales assessing positive affect and negative affect. PANAS items are rated on 6-point Likert scale (from 1 “*do not feel*” to 6 “*feel very strongly*”). In this study, we used the Japanese version of PANAS (PANAS-J) developed by Sato and Yasuda [40]. Its reliability and validity has been confirmed [40]. The factor structure of the PANAS-J was identical to the original version. In this study, the internal consistencies of the PANAS-J subscales were both adequate (Cronbach’s α = 0.89 for both).

### Data Analysis

We randomly divided the combined samples into 2 subsample groups to ensure mutual independence of the samples used for two different factor analyses. The first subsample group including 486 participants (199 males and 287 females; mean age = 21.87, SD = 8.19) was used for exploratory factor analysis. The second subsample group including 487 participants (230 males and 257 females; mean age = 21.40, SD = 6.67) was used for confirmatory factor analysis.

We conducted a factor analysis using the maximum likelihood method with the oblimin rotation to specify a statistically appropriate factor structure. The number of factors was decided based on a scree plot and parallel analysis. We conducted a confirmatory factor analysis with a maximum likelihood estimation to investigate the model fitness of the factor structure found by the exploratory factor analysis, and to choose the most well-fitted factor structure by comparing the following models: the factor structure found by the exploratory factor analysis in this study (16 items, single factors model; 16 items, original 2 factors model [24]; 16 items, another 2 factor model [25]; and 12 items, 2 factors model [27]). Goodness-of-fit indices including the standardized root-mean residual, the root mean square error of approximation, the Comparative Fit Index, and the Tucker-Lewis Index were examined to determine how well the model fit the data. Following the existing recommendations [41–45], indices such as standardized root-mean residual < 0.08, root mean square error of approximation < 0.08, Comparative Fit Index > .95, and Tucker-Lewis Index > .95 were used to compare the DPSS-R-J models.

**Fig 2:**
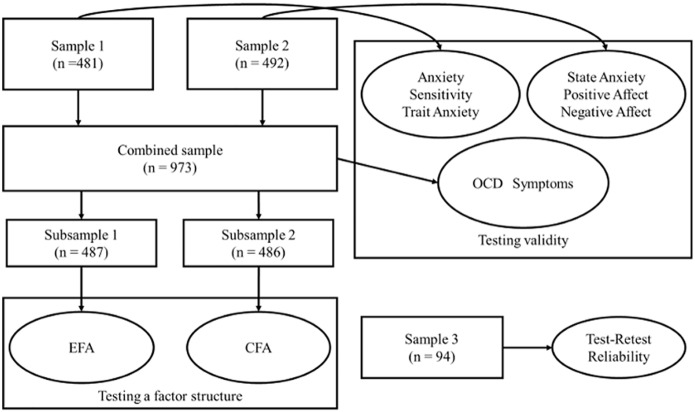
Relationship between the samples and the analyses in this study. CFA: confirmatory factor analysis; EFA: exploratory factor analysis; OCD: obsessive-compulsive disorder.

The reliability of the DPSS-R-J was tested using Cronbach’s alpha coefficient and test-retest intra-class correlation coefficients. We computed intraclass correlation coefficients using the Sample 3 dataset in this study. To examine the validity of the DPSS-R-J, we computed correlation coefficients between DPSS-R-J subscales, obsessive-compulsive symptoms (PI-J), emotional traits (STAI-T-J and ASI-J), emotional states (STAI-S-J and PANAS-J). We used datasets for the estimations in the following manner: Sample 1 was used to investigate the relationship between DPSS-R-J subscales and emotional traits, Sample 2 was used to investigate the relationship between DPSS-R-J subscales and emotional states, and the combined sample was used to investigate the relationship between DPSS-R-J and obsessive-compulsive symptoms. For convergent validity, it is expected that the disgust propensity and disgust sensitivity subscales of the DPSS-R-J are strongly correlated with STAI-T-J, ASI-J, and PI-J subscales. Especially for PI-J, the dirt subscale is expected to correlate with disgust propensity and disgust sensitivity stronger than other PI-J subscales. Moreover, DPSS-R-J subscales are expected to correlate with STAI-S-J and the negative-affect subscale of PANAS-J moderately because DPSS-R-J is regarded as a measure of trait disgust rather than state disgust. For discriminant validity, we expected that the DPSS-R-J subscales are would not be correlated with the positive-affect subscale of PANAS-J. Fig 2 illustrates the relationships between the samples and the analyses in this study.

## Results

In this study, all analyses were performed using the R version 3.0.2 (R Development Core Team, Vienna, Austria) software package. Of the 108976 responses, there were only 93 missing values (0.09%) in the dataset. Missing values were completed by the multiple imputation method using the “mi” package for the R [46].

### Factor Analysis

The Kaiser-Meyer-Olkin measure of sampling adequacy was .93, and measures of sampling adequacy for each item ranged from .89 to .96. In addition, Bartlett's test of sphericity was significant at *p* < .001. These measures indicated that the data set was appropriate for factor analysis.

To specify the underlying factor structure of DPSS-R-J, we conducted an exploratory factor analysis (maximum likelihood estimation, oblimin rotation) for the 16 original items. Based on the scree plot (first four eigenvalues were 6.265, 1.366, 1.055, and 0.979) and the parallel analysis, two factors were established that accounted for 46.15% of the total variance. Table 1 shows the factor loadings of the DPSS-R-J. Exclusion criteria for the factor loading of each item was a score below .30, as was the case in a previous study [24]. No item was discarded because of factor loadings above .30. This factor structure was identical to that of the original version of the scale [24]. Therefore, we regarded the first factor as the disgust propensity subscale and the second factor as the disgust sensitivity subscale. The 2 factors were positively correlated (*r* = .67).

**Table 1:** Exploratory factor analysis of the DPSS-R-J.

Item		Factor 1	Factor 2	h^2^
10.	I experience disgust.	**.93**	-.20	.90
5.	I feel repulsed.	**.78**	-.03	.61
14.	I find something disgusting.	**.67**	.04	.44
12.	I become disgusted more easily than other people.	**.66**	.09	.45
9.	When I experience disgust, it is an intense feeling.	**.63**	.09	.40
7.	I screw up my face in disgust.	**.51**	.09	.27
1.	I avoid disgusting thing.	**.42**	.09	.18
6.	Disgusting things make my stomach turn.	**.35**	.29	.21
11.	It scares me when I feel faint.	-.09	**.70**	.50
3.	It scares me when I feel nauseous.	-.06	**.68**	.46
8.	When I notice that I feel nauseous, I worry about vomiting.	.08	**.52**	.27
4.	I think disgusting items could cause me illness / infection.	.07	**.51**	.26
16.	I think feeling disgust is bad for me.	.03	**.50**	.25
2.	When I feel disgusted, I worry that I might pass out.	.00	**.47**	.22
15.	It embarrasses me when I feel disgusted.	.23	**.45**	.26
13.	I worry that I might swallow a disgusting thing.	.27	**.38**	.22

Factor loadings in bold denote meeting the criteria (> .30).

Next, we compared the possible models of the DPSS-R-J factor structure with a confirmatory factor analysis for the combined sample. As shown in Table 2, a confirmatory factor analysis revealed that fitness indices of the factor structure found by the exploratory factor analysis were statistically acceptable (Comparative Fit Index = .92, Tucker-Lewis Index = .90, root mean square error of approximation = .07, and standardized root-mean residual = .05), and most of the indices indicated the model derived from the exploratory factor analysis in this study was better fitted compared to other factor structures. Above all, we decided to adopt this model as the Japanese version of DPSS-R.

**Table 2:** Goodness of fit in the models.

		GFI	AGFI	CFI	TLI	RMSEA	SRMR
1.	16 items, 2-factor model (identified in the EFA)	.92	.90	.92	.90	.07	.05
2.	16 items, single-factor model	.87	.83	.85	.83	.09	.07
3.	16 items, 2-factor model (Olatunji et al., 2007)	.88	.84	.87	.85	.09	.06
4.	12 items, 2-factor model (Fergus & Valentiner, 2009)	.93	.90	.92	.90	.08	.06

GFI: Goodness of Fit Index; AGFI: Adjusted Goodness of Fit Index; CFI: Comparative Fit Index; TLI: Tucker-Lewis Index; RMSEA: Root Mean Square Error of Approximation; SRMR: Standardized Root-Mean Residual. The model number 1, which was identified in the EFA, is identical to that of the original version of the DPSS-R (van Overveld et al., 2006).

### Descriptive Statistics

Table 3 shows the descriptive statistics of the variables in this study. Statistical values for disgust propensity and disgust sensitivity were based on the aforementioned results from factor analyses. T-tests were conducted to examine gender differences in disgust propensity and sensitivity. There were no significant gender differences for any of these variables (*t* = 0.03, *p* = .98; *t* = 0.01, *p* = 99, respectively).

**Table 3:** Descriptive statistics of the variables.

				95% CI		
	Mean	SD	SE	upper	lower	Range
Sample 1 (n = 481)						
DP	2.72	0.83	0.04	2.79	2.64	1.13–5.38
DS	2.19	0.78	0.04	2.26	2.20	1.13–5.50
ASI-J	0.95	0.66	0.03	1.01	0.89	0.00–3.75
STAI-T-J	2.15	0.50	0.02	2.20	2.11	0.40–3.50
Sample 2 (n = 492)						
DP	3.03	0.79	0.04	3.01	2.96	1.38–5.38
DS	2.55	0.76	0.03	2.67	2.52	1.25–5.50
PANAS-J-Positive Affect	2.58	0.96	0.04	2.66	2.50	1.00–5.50
PANAS-J-Negative Affect	2.16	0.94	0.04	2.24	2.08	1.00–4.75
STAI-S-J	2.15	0.41	0.02	2.19	2.11	1.30–3.45
Combined sample (n = 973)						
DP	2.88	0.77	0.03	2.29	2.83	1.25–5.25
DS	2.28	0.73	0.02	2.32	2.23	1.13–5.38
PI-J-Dirt	1.12	0.70	0.02	1.24	1.15	0.00–3.91
PI-J-Check	1.15	0.82	0.03	1.20	1.09	0.00–4.00
PI-J-Precision	0.60	0.75	0.02	0.65	0.56	0.00–3.83
PI-J-Impulse	0.52	0.68	0.02	0.56	0.48	0.00–4.00
PI-J-Doubt	1.13	0.79	0.03	1.35	1.25	0.00–3.94
PI-J-Total	1.01	0.61	0.02	1.05	1.05	0.00–3.74
Sample 3 (n = 93)						
Time 1—DP	2.46	0.68	0.07	2.60	2.33	1.25–4.25
Time 2—DP	2.51	0.71	0.07	2.66	2.36	1.00–4.50
Time 1—DS	1.99	0.66	0.07	2.12	1.85	1.00–4.50
Time 2—DS	2.11	0.79	0.08	2.27	1.95	1.00–4.63

SD: Standard Deviation; SE: Standard Error; 95% CI: 95% Confidence Interval; DP: Disgust Propensity; DS: Disgust Sensitivity; ASI-J; Japanese version of the Anxiety Sensitivity Index; STAI-T-J; Japanese version of the State Trait Anxiety Inventory-Trait; PANAS-J: Japanese version of the Positive and Negative Affect Schedule; STAI-S-J: Japanese version of the State Trait Anxiety Inventory-State; PI-J: Japanese version of the Padua Inventory.

### Reliability

The Cronbach's alpha of the disgust propensity and disgust sensitivity subscales were 0.86 and 0.79, respectively. The test-retest intraclass correlation coefficient with four weeks intervals was 0.72, 95% CI = 0.60 to 0.80 for disgust propensity and 0.75, 95% CI = 0.65 to 0.83 for disgust sensitivity. These results provided empirical support for the reliability of the DPSS-R-J.

### Validity

We calculated correlation coefficients of DPSS subscales with measures of convergent validity (STAI-T-J, ASI-J, PI-J, STAI-S-J, and PANAS-J-Negative Affect) and discriminant validity (PANAS-J-Positive Affect). The results are displayed in Table 4.

**Table 4:** DPSS-R-J correlations with convergent and discriminant measures.

	DP	DS
sample 1 (n = 481)		
ASI-J	**.52**	**.66**
STAI-T-J	**.51**	**.44**
sample 2 (n = 492)		
PANAS-J-Positive Affect	.00	.05
PANAS-J-Negative Affect	**.34**	**.34**
STAI-S-J	**.33**	**.38**
combined sample (n = 973)		
PI-J-Dirt	**.46**	**.45**
PI-J-Check	**.39**	**.41**
PI-J-Precision	**.29**	**.31**
PI-J-Impulse	**.31**	**.32**
PI-J-Doubt	**.54**	**.54**
PI-J-Total	**.53**	**.53**

Correlations in bold denote statistical significance with *p* < .05. DP: Disgust Propensity; DS: Disgust Sensitivity; ASI-J: Japanese version of the Anxiety Sensitivity Index; STAI-T-J: Japanese version of the State-Trait Anxiety Inventory-Trait; STAI-S-J: Japanese version of the State-Trait Anxiety Inventory-State; PANAS-J: Japanese version of the Positive and Negative Affect Schedule; PI-J: Japanese version of the Padua Inventory.

These results were generally in accordance with our expectations; however, disgust propensity and disgust sensitivity showed similar trends in relation to the correlational coefficients. This implies both concepts substantially overlap each other. If so, the conceptual independence of disgust propensity and disgust sensitivity is doubtful. To examine this issue, we conducted a series of multiple regression analyses; the ASI-J, STAI, PANAS, and PI scores were regressed according to the disgust propensity and sensitivity subscales of the DPSS-R-J. The results are displayed in Table 5. The predictive effects of disgust propensity and sensitivity on the PI subscales, PANAS-NA, and STAI-S scores were both statistically significant, even after controlling for each other. These results indicate that disgust propensity and disgust sensitivity each have a unique effect on obsessive-compulsive symptoms, state anxiety, and negative affectivity. Moreover, consistent with our hypotheses, ASI was better predicted by disgust sensitivity than disgust propensity, and STAI-T was better predicted by disgust propensity than disgust sensitivity. These results indicate disgust propensity and disgust sensitivity are conceptually distinguishable, at least in this regard. From the above, we can conclude that empirical support for the construct validity of the DPSS-R-J was obtained.

**Table 5:** Standardized regression coefficients of disgust propensity and disgust sensitivity on convergent and discriminant measures.

	DP		DS	
	β	95% CI	Β	95% CI
sample 1 (n = 481)				
ASI-J	**.16**	.07 to .24	**.55**	.46 to .64
STAI-T-J	**.39**	.28 to .49	**.19**	.09 to .29
sample 2 (n = 492)				
PANAS-J-Positive Affect	-.06	-.18 to .05	.06	-.05 to .18
PANAS-J-Negative Affect	**.16**	.06 to .27	**.27**	.17 to .37
STAI-S-J	**.19**	.09 to .30	**.20**	.10 to .31
combined sample (n = 973)				
PI-J-Dirt	**.29**	.22 to .36	**.27**	.20 to .34
PI-J-Check	**.22**	.15 to .30	**.26**	.19 to .34
PI-J-Precision	**.15**	.07 to .23	**.21**	.13 to .29
PI-J-Impulse	**.18**	.11 to .26	**.20**	.12 to .28
PI-J-Doubt	**.32**	.26 to .39	**.33**	.27 to .40
PI-J-Total	**.31**	.25 to .38	**.33**	.26 to .40

β coefficients in bold denote statistical significance with *p* < .05. β: standardized regression coefficient; 95% CI: 95% Confidence Interval; DP: Disgust Propensity; DS: Disgust Sensitivity; ASI-J: Japanese version of the Anxiety Sensitivity Index; STAI-T-J: Japanese version of the State-Trait Anxiety Inventory-Trait; STAI-S-J: Japanese version of the State-Trait Anxiety Inventory-State; PANAS-J: Japanese version of the Positive and Negative Affect Schedule; PI-J: Japanese version of the Padua Inventory.

## Discussion

The purpose of this study was to develop a Japanese version of the revised disgust propensity and sensitivity scale. To achieve this, we specified factors of the DPSS-R-J and evaluated its reliability and construct validity. We found that the factor structure of the DPSS-R-J that included the 2 subscales (disgust propensity and disgust sensitivity) was identical to that of the original study. Correlation and multiple regression analyses indicated that subscales of the DPSS-R-J had acceptable convergent and discriminant validity. Furthermore, the DPSS-R-J was found to have good internal consistency and acceptable test-retest reliability.

### Factor Structure

This study replicated the bidimensional factor structure (disgust propensity and disgust sensitivity) of the DPSS-R using a sample from the Japanese population. It provided empirical support for conceptual distinction between disgust sensitivity and propensity, as well as factorial validity of the DPSS-R-J. The results of the confirmatory factor analysis showed that the item-factor structure of the DPSS-R-J identified in the EFA including 16 items and 2 factors was statistically more adequate than were the others. In particular, the fitness indices of the 12-item factor structure were comparable to the factor structure identified in the EFA, suggesting it may be possible to establish the short version of the DPSS-R-J using 12 items. Future research is needed to examine the reliability and validity of the shortened version of the DPSS-R-J.

In this study, the majority of the participants were aged approximately 20 years. This age bias endangers the representativeness of the sample; therefore, one should be cautious when generalizing the factor structure of the DPSS-R-J to all Japanese age groups. In future research, the factor structure should be cross-validated using samples with a wider age range.

### Reliability and Validity

We examined the reliability of DPSS-R-J subscales to estimate Cronbach’s alpha coefficient as a parameter of internal consistency, and intra-class correlation coefficient as a parameter of test-retest reliability. Results showed that reliability of DPSS-R-J is comparable to the original DPSS-R.

The convergent and discriminant validity of the DPSS-R-J were supported by the following correlation and multiple regression analyses results. (1) Disgust sensitivity and propensity were both positively correlated with trait anxiety and anxiety sensitivity, as predicted in our Hypothesis 1. (2) Disgust sensitivity and propensity positively correlated with state anxiety and negative affect, as predicted in our Hypothesis 2. (3) Similar to past research [26], disgust sensitivity related more closely with anxiety sensitivity than trait anxiety, as predicted in Hypothesis 3 (*β* = .55 versus .16). (4) On the contrary, disgust propensity related more closely with trait anxiety than anxiety sensitivity, as predicted in our Hypothesis 4 (*β* = .39 versus .19). (5) Disgust propensity and sensitivity showed no significant association with positive affect (*r* = .00 and .05, respectively), as predicted in our Hypothesis 5. (6) Disgust propensity and sensitivity positively correlated with all subscales of the PI-J, as we predicted. In addition, the predictive effects of disgust propensity and sensitivity on PI-J were statistically significant, even controlling for each other; however, both DPSS-R-J subscales correlated most strongly with the doubt subscale of the PI, in contrast to our Hypothesis 6.

Basically, these results support the construct validity of the DPSS-R-J. Regarding Hypothesis 1, numerous studies have indicated that anxiety and disgust are distinct but correlated emotional constructs [47, 48]. Similar to the background literature, the results of this study showed that the emotional traits relevant to disgust and anxiety were moderately correlated with each other. Concerning Hypotheses 2 and 5, these results showed that disgust propensity and sensitivity were related to negative emotional states; however, they were not related to positive emotional states, which is consistent with the findings of Olatunji et al. [25]. The results of multiple regression analyses provided empirical support for Hypotheses 3 and 4, suggesting that disgust propensity and disgust sensitivity are at least partially conceptually distinguishable. The correlation and multiple regression analyses provided mixed results for Hypothesis 6. Disgust propensity and sensitivity positively correlated with obsessive-compulsive symptoms; moreover, both had unique predictive effects on these symptoms. These results provide empirical support for the construct validity of DPSS-R-J and the conceptual independence of its subscales. On the other hand, the DPSS-R-J subscales were more closely correlated to the doubt subscale than the dirt subscale of the PI-J. According to a previous study [49], chronic doubting is a common cognitive characteristic of individuals with obsessive-compulsive disorder. Regardless of the symptoms, most individuals with obsessive-compulsive disorder will describe a feeling of uncertainty regarding their own behavior, and this in turn results in repetitive actions. This implies that doubting has a cognitive effect on various obsessive-compulsive disorder symptoms. From this standpoint, the results of this study imply that disgust traits are related to the cognitive-based obsessive-compulsive disorder symptoms. This implication should be tested in future research.

### Limitations

This study has several limitations. First, there were some biases in the selection of participants. As mentioned above, the age distribution is biased limitation. Furthermore, we did not control for participants’ socio-economic status. In addition, we did not conduct formal assessments of psychopathology and merely relied on participants’ self-reported history of mental illnesses. Consequently, the influence of age, socio-economic status, and psychopathology were not adequately controlled. This limitation affects the generalizability of our findings. Second, participants in this study did not include a clinical population. Considering the relationship between disgust and psychopathology, the psychometric properties of DPSS-R-J in samples with medical diseases should be investigated with future research. Third, there were no gender differences for the DPSS-R-J scores in this study, although these have consistently been found in previous studies [25, 29]. This may have been due to the aforementioned sampling bias, a psychometric characteristic of the DPSS-R-J itself, or a culturally specific Japanese characteristic of disgust. The results of this study do not allow us to make a definite conclusion concerning this issue, therefore, it should be addressed in future research. In addition, the conceptual distinction between disgust propensity and disgust sensitivity was supported by the results of multiple regression analyses; however, the correlational trends for these two concepts were quite similar, specifically concerning the obsessive-compulsive symptoms. This suggests that the conceptual uniqueness of the two disgust traits assessed by the DPSS-R-J is still unclear. Therefore, further examination of this issue is necessary.

## Conclusion

We examined the factor structure, reliability, and validity of the DPSS-R-J. The item-factor structure of the DPSS-R-J was identical to that of its original English version (DPSS-R). The DPSS-R-J comprised two subscales: disgust propensity and disgust sensitivity. Both subscales had adequate internal consistency and test-retest reliability. The results of this study demonstrated the DPSS-R-J’s construct validity. Although there were some limitations, this study provided the first examples of empirical support for the DPSS-R-J.

## Supporting Information

S1 Table Datasets. (XLSX)

S2 Table Items of DPSS-R-J. (XLSX)

S3 Table Items of DPSS-R. (XLSX)
